# Dynamic properties of enhancer and promoter during DNA damage in hepatocellular carcinoma

**DOI:** 10.1016/j.isci.2025.112565

**Published:** 2025-05-02

**Authors:** Jinyuan Zhang, Tianyu Ma, Longjun Xian, Zhiyun Guo

**Affiliations:** 1 School of Life Sciences and Engineering, Southwest Jiaotong University, Chengdu 610031, China; 2 Department of Thoracic Surgery, Institute of Thoracic Oncology, Frontiers Science Center for Disease-related Molecular Network, West China Hospital, Sichuan University, Chengdu 610213, China

**Keywords:** Molecular biology, Cancer, Genomics

## Abstract

DNA damage is a critical factor contributing to tumorigenesis, however, the dynamic changes in multi-omics signatures of enhancers and promoters during the DNA damage response (DDR) remain poorly understood. In this study, we discovered that the expression levels, chromatin accessibility, and element activity of distal/proximal enhancers and promoters exhibited obvious dynamic similarity and duality of characteristics at different stages of DNA damage in hepatocellular carcinoma (HCC). Furthermore, we found that the pre-damage accessibility and activity status of enhancers and promoters played an important role in determining their regulatory features following DNA damage in HCC. Finally, we identified transcription factors (TFs) with significantly altered activity in response to DNA damage, with notable differences between p53 binding activity in enhancers and promoters during the DDR. Overall, these findings reveal the complex dynamic changes within *cis*-regulatory elements in response to DNA damage.

## Highlights

• Enhancers and promoters exhibit dynamic characteristic changes during DNA damage
• Pre-damage chromatin states affect how enhancers and promoters respond to DNA damage
• Enhancers and promoters display different p53 binding activity after DNA damage

## Graphical abstract

**Figure:**
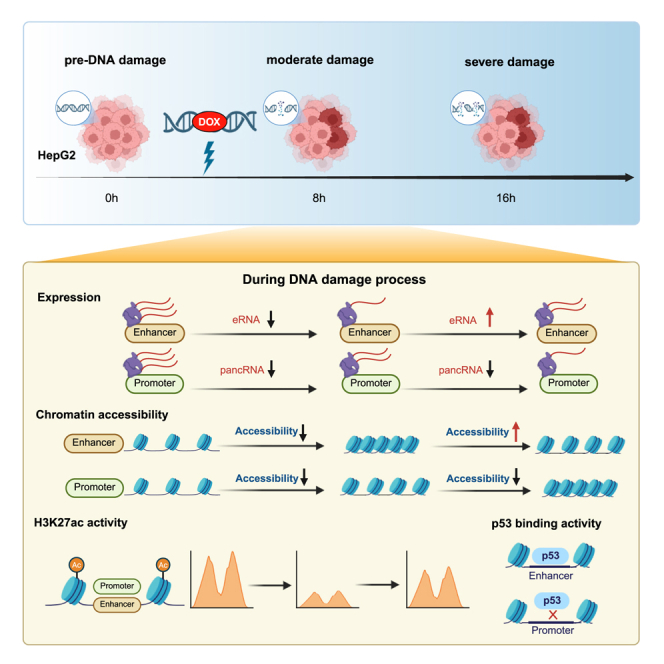

## Introduction

As *cis*-regulatory elements (CREs), enhancers and promoters often collaborate to exert their crucial regulatory functions in various diseases, such as cancer.^1^ Although enhancers and promoters have long been regarded as two distinct regulatory elements, recent research suggests that the boundary between them is gradually becoming blurred, and they often exhibit similar properties and functions. For example, some promoters could exhibit enhancer-like activity to perform enhancer function, and conversely, some activated enhancers can initiate local transcription at their boundaries, thereby functioning as promoters.^2^^,^^3^^,^^4^ Furthermore, previous studies have demonstrated that both enhancers and promoters can transcribe short, unspliced RNAs, such as enhancer RNAs (eRNAs)^5^ and promoter-associated noncoding RNAs (pancRNAs).^6^^,^^7^ These findings of differences and similarities indicate that defining enhancers and promoters as two entirely distinct regulatory elements is increasingly being challenged, and they are far more complex than we currently understand.

DNA can be damaged under various stresses, triggering DNA damage response (DDR) that aims to maintain the genomic integrity and stability of DNA, this regulatory mechanism is often vital for cell survival and physiological functions.^8^ Enhancers and promoters are not only pivotal in gene regulation but also interact with the processes of DNA damage and repair. They are typically located in regions of accessible chromatin, this state makes them more susceptible to DNA damage, such as double-strand breaks (DSBs).^9^ Previous studies have shown that enhancers and promoters recruit DNA repair proteins and regulatory factors during the DDR, affecting their activity to regulated genes involved in the DDR.^10^ The dysregulation of DDR is associated with hepatocellular carcinoma (HCC) and affects responses to DNA-damaging anticancer therapy.^11^ Nevertheless, how the regulatory features of enhancers and promoters dynamically change in response to DNA damage, what the similarities and differences they exhibit during DNA damage, and how these changes impact the development and progression of HCC remain unclear.

In this study, we treated HCC HepG2 cells with doxorubicin (DOX) for 0 h, 8 h, and 16 h to represent different stages of DNA damage (pre-damage, moderate damage, and severe damage), and performed RNA-seq, ATAC-seq, and H3K27ac ChIP-seq to assess the dynamic changes in the transcriptional regulation of distal/proximal enhancers and promoters. The results showed that the expression levels, chromatin accessibility, and activity exhibited significantly different distribution characteristics and trends of change among these *CREs* during DNA damage. We also found that the pre-damage accessibility states and activity states of distal/proximal enhancers and promoters significantly influenced their post-damage characteristic changes. Furthermore, the vast majority of enhancer and promoter regions remained in a “consistently accessible” and “consistently active” state during DNA damage. Analysis of the correlations between various chromatin states in enhancer and promoter regions during DNA damage revealed that high levels of expression, accessibility, and activity of enhancers and promoters are often tightly associated during DNA damage. Ultimately, we discovered that transcription factors (TFs) exhibit significant activity changes during DNA damage, and notably, p53 leads to property changes in enhancer and promoter regions. Overall, these findings provide a novel comprehensive resource that reveals the intricate dynamical changes of chromatin within regulatory element regions in response to DNA damage, highlighting the distinct patterns of change among various regulatory elements.

## Results and discussion

### Dynamic changes of enhancer and promoter expression profiles in the HepG2 cells during DNA damage

**Figure 1:**
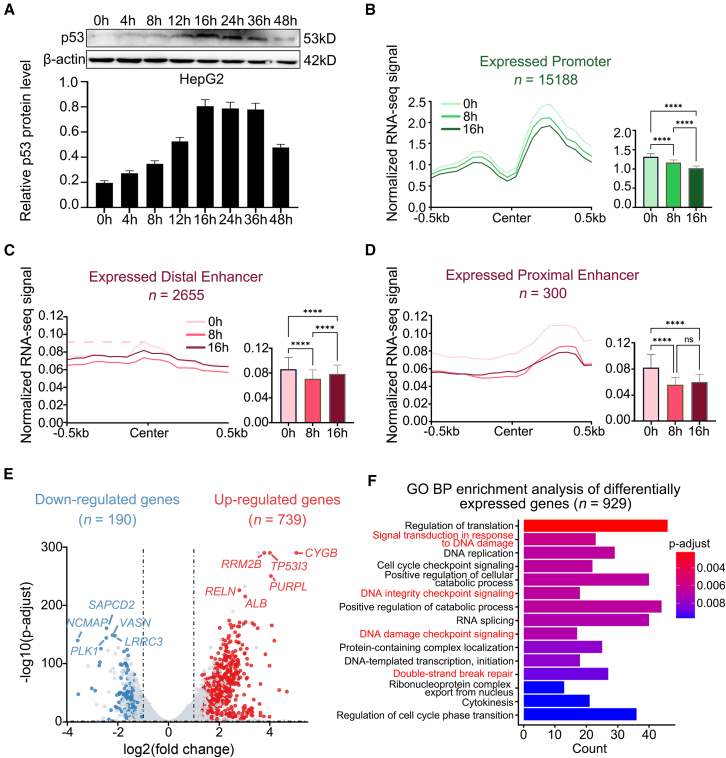
Dynamic changes in transcriptional expression during DNA damage (A) Western blot analysis showing the expression of p53 protein in HepG2 cells treated with DOX from 0h to 48h. β-actin serves as the control. (B–D) Average RNA-seq signal profiles in HepG2 cells treated with DOX at 0 h, 8 h, and 16 h for promoters, distal enhancers, and proximal enhancers (left panels). 0 h, 8 h, and 16 h indicate pre-damage, moderate damage, and severe damage, respectively. The midpoint of the promoter and enhancer regions is labeled as “Center”. Numbers above plots indicate counts of elements for this condition. Quantification of RNA-seq signals represented as bar plots of reads per million (RPM) (right panels). Data are presented as mean ± SEM. Statistical significance was assessed using paired non-parametric Friedman tests. ∗*p* < 0.05, ∗∗*p* < 0.01, ∗∗∗*p* < 0.001, ∗∗∗∗*p* < 0.0001. (E) Volcano plot showing differentially expressed genes at 0 h, 8 h, and 16 h with consistent upregulation or downregulation. Criteria: |log2(fold change) | > 1, FDR <0.05. The genes with the most significant differential expression have been annotated. (F) GO BP enrichment analysis of the top 15 pathways for the 929 consistency of upregulated or downregulated expressed genes. The four DNA damage-related pathways have been highlighted in red.

Previous studies have reported that the extent of DNA damage increases progressively with the duration of DOX treatment.^12^^,^^13^^,^^14^ To obtain HCC cells at different levels of DNA damage, HepG2 cells were treated with DOX for 0, 4, 8, 12, 16, 24, 36, and 48 h. Existing researches has indicated a correlation between p53 protein levels and the capacity for DNA damage repair.^15^^,^^16^ Furthermore, p53 is recognized as a pivotal protein in modulating enhancers and promoters during the DDR. Consequently, we performed western blot analysis to determine p53 protein levels. Results showed that p53 protein expression initially increased, peaking at 16 h, and then decreased (Figure 1A). Therefore, based on the progressive intensification of DNA damage with DOX treatment duration and the corresponding changes in p53 protein expression, we ultimately selected the three time points 0 h, 8 h, and 16 h to represent different stages of DNA damage (pre-damage, moderate damage, and severe damage, respectively) to investigate the dynamic changes in the regulatory mechanisms of HepG2 cells in response to DNA damage.

ERNAs, which are believed to play a crucial role in enhancer activity and target gene regulation.^5^^,^^17^ Similarly, promoter regions can transcribe pancRNAs, which can influence downstream gene expression through interactions with TFs and other regulatory proteins,^6^^,^^7^ however, the dynamic changes in the transcription levels of enhancers and promoters in HCC after DNA damage are not well understood. To address this, we first downloaded data for 47,651 enhancers and 15,974 promoters in HepG2 cells from the ENCODE.^2^ To eliminate interference from gene transcripts on enhancer regions, we removed enhancers overlapping with gene coding regions, as a result, 7,631 enhancers were ultimately obtained for further study (Table S1). Next, we performed RNA-seq on HepG2 cells treated with DOX at 0 h, 8 h, and 16 h to detect the expression levels of promoters and enhancers under different levels of DNA damage. After filtering out non-expressing regions of enhancer and promoter, we identified 15,188 expressed promoters and 2,955 expressed enhancers (Table S1).

The results indicated that the expression around the promoter center regions exhibited an asymmetric bimodal distribution, with expression levels showing a significant trend of decrease as DNA damage progressively increased (Figure 1B). Previous studies have shown that proximal and distal enhancers have significantly different regulatory mechanisms, impacting tumorigenesis and progression.^18^^,^^19^ Therefore, we aimed to explore the similarities and differences in transcriptional expression patterns of proximal and distal enhancers following DNA damage. Based on ENCODE annotations, we classified the 2,955 expressed enhancers into 2,655 distal enhancers and 300 proximal enhancers. We found that the expression levels of distal enhancers were highest at the central position compared to the flanking regions, with expression significantly decreasing and then significantly increasing during three different levels of DNA damage (Figure 1C). In contrast, proximal enhancers, similar to promoters, showed a continuous decrease in expression during DNA damage, suggesting that both transcripts of proximal enhancers and promoters are not primarily involved in DNA damage repair but rather exhibit reduced expression activity following DNA damage (Figure 1D).

We expect an increase/decrease in the expression of genes involved in DDR as a result of prolonged DNA damage. Therefore, we further screened 929 genes (739 upregulated and 190 downregulated) that were consistently upregulated or downregulated across three time points (0 h, 8 h, and 16 h) during DNA damage (Figure 1E; Table S2). GOBP enrichment analysis of these genes indicated significant enrichment in DDR-related pathways (Figures 1F and S1), and gene set enrichment analysis (GSEA) analysis indicated that most of the genes involved in these pathways are upregulated after DNA damage (Figure S2). In summary, we found distinctly different transcriptional expression changes among distal enhancers, proximal enhancers, and promoters during DNA damage. Notably, the expression of distal enhancers significantly decreases and then increases during DNA damage, suggesting that distal eRNAs are principal regulatory factors in the DDR.

### Chromatin accessibility of enhancer and promoter during DNA damage

**Figure 2:**
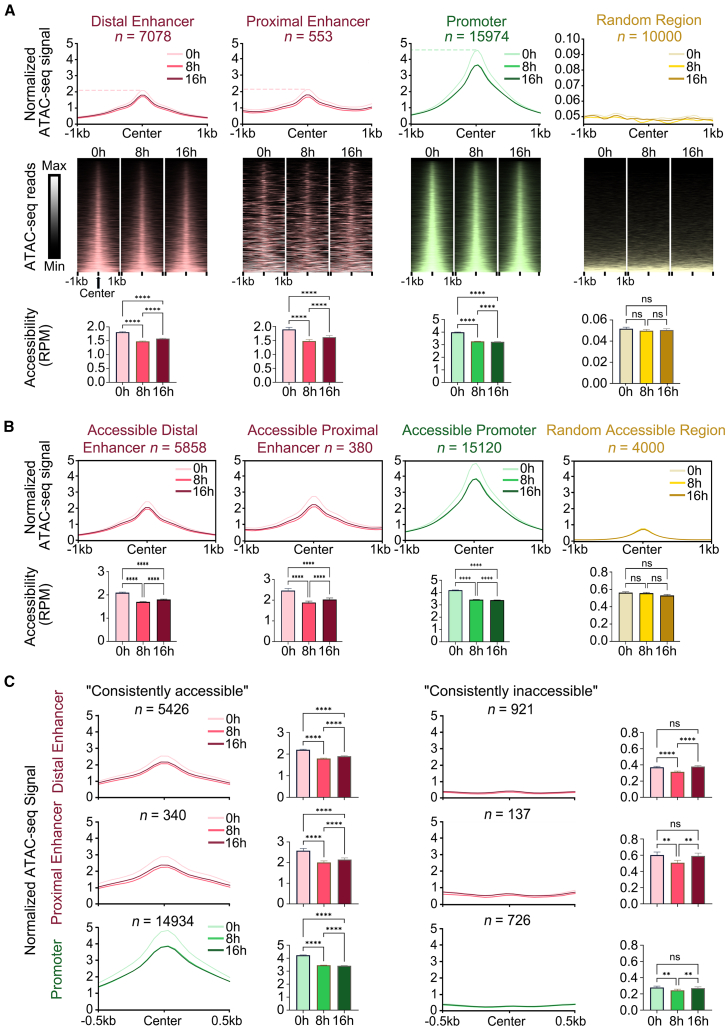
Dynamic changes in chromatin accessibility during DNA damage (A) Average ATAC-seq signal profiles within 1kb around the center of distal enhancers, proximal enhancers, promoters, and random genomic regions at 0 h, 8 h, and 16 h (top panels). Heatmap of ATAC-seq reads density within 1kb around the center of distal enhancers, proximal enhancers, promoters, and random genomic regions at 0 h, 8 h, and 16 h (mid panels). Quantification of ATAC-seq signals represented as bar plots (bottom panels). (B) Average ATAC-seq signal profiles of accessible distal enhancers, proximal enhancers, promoters, and random accessible regions (top panels). Quantification of ATAC-seq signals represented as bar plots (bottom panels). “Random accessible regions” were randomly selected from the ATAC peaks, excluding those overlapping with enhancer and promoter regions. (C) Average ATAC-seq signal profiles for “consistently accessible” and “consistently inaccessible” states of distal enhancers, proximal enhancers, and promoters. Quantification of ATAC-seq signals represented as bar plots. Numbers above plots indicate counts of elements for this condition. Data are presented as mean ± SEM. Statistical significance was assessed using paired non-parametric Friedman tests. ∗*p* < 0.05, ∗∗*p* < 0.01, ∗∗∗*p* < 0.001, ∗∗∗∗*p* < 0.0001.

Subsequently, we performed ATAC-seq on HepG2 cells treated with DOX at 0 h, 8 h, and 16 h to evaluate the dynamic changes in chromatin accessibility of enhancers and promoters under different degrees of DNA damage (pre-damage, moderate damage, and severe damage) (Table S3). It is found that, following DNA damage, the chromatin accessibility of both proximal and distal enhancer regions showed a significant decrease followed by an increase, compared to the chromatin accessibility of random regions, which did not show significant changes (Figure 2A). Based on previous research, we propose that this trend reflects the process of chromatin condensation as a temporary protective mechanism against DNA damage in the initial stages of DNA damage, and subsequently decompresses to increase chromatin accessibility for enhancers to facilitate DDR.^20^^,^^21^ Previous study has shown that eRNA expression relies on the chromatin accessibility of enhancer regions,^22^ and considering that distal enhancer eRNA expression levels exhibit an initial decrease followed by an increase (Figure 1C), these observations imply a coordinated alteration between eRNA expression and chromatin accessibility during DNA damage. This indicates that DNA damage may influence the expression of distal enhancer eRNAs by modulating the chromatin accessibility. Conversely, the chromatin accessibility of promoter regions continues to decrease, suggesting that DNA damage persistently inhibits transcriptional regulation of promoters (Figure 2A). Furthermore, promoter regions consistently exhibited higher ATAC-seq signal levels compared to distal/proximal enhancer regions (Figure S3).

To investigate whether enhancers and promoters with varying initial accessibility states (accessible and inaccessible) are differentially affected by DNA damage, we classified distal/proximal enhancers and promoters into accessible and inaccessible elements based on pre-damage (0 h) accessibility states (Table S3). We then compared the chromatin accessibility levels of these elements during DNA damage (0 h, 8 h, and 16 h). The results showed that both accessible enhancers and promoters exhibited a decreasing trend in chromatin accessibility following DNA damage, whereas there was no significant change in the random accessible regions (Figure 2B). In contrast, both inaccessible enhancers and promoters increased with chromatin accessibility after DNA damage (8 h and 16 h) (Figure S4), suggesting that these enhancers and promoters might participate in the DDR process by increasing their chromatin accessibility.

To further compare the dynamic changes in chromatin accessibility levels of enhancer and promoter regions under DNA damage, we further subdivided the accessible/inaccessible enhancers and promoters at pre-damage (0 h) accessibility states into two categories: “consistently accessible” element regions, which are accessible at all time points (0 h, 8 h, and 16 h) and “consistently inaccessible” element regions, which are inaccessible at all time points (0 h, 8 h, and 16 h). We found that the vast majority of enhancer (distal: 77%; proximal: 61%) and promoter (93%) regions remained in a “consistently accessible” state during DNA damage (Table S3). Additionally, the chromatin accessibility levels of “consistently accessible” regions exhibited a significant decrease after DNA damage (Figure 2C). Conversely, the “consistently inaccessible” regions were not significantly affected by DNA damage (Figure 2C), indicating that chromatin accessibility of *CREs* is a dominant factor to perform regulatory function in responding to DNA damage. In summary, the chromatin accessibility levels of enhancers and promoters show significantly different changing trends during the DNA damage process, and the pre-damage accessibility states of enhancers and promoters lead to significantly different changes in their post-damage accessibility levels.

### Histone modification of enhancer and promoter during DNA damage

**Figure 3:**
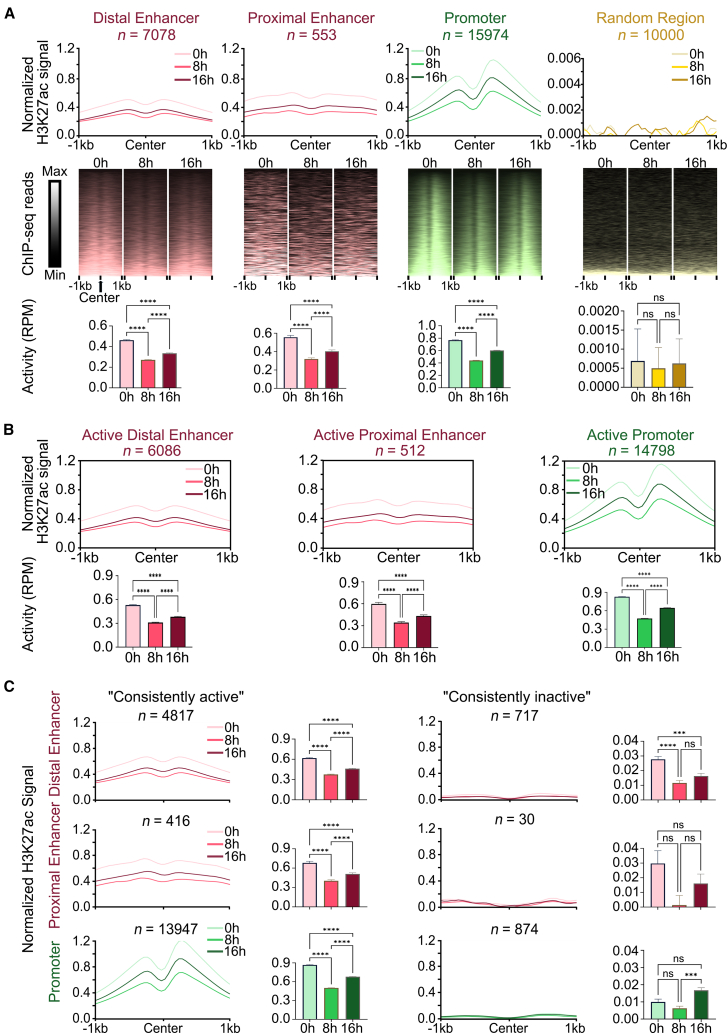
Dynamic changes in histone modification (H3K27ac) during DNA damage (A) Average H3K27ac signal profiles within 1kb around the center of distal enhancers, proximal enhancers, promoters, and random genomic regions at 0 h, 8 h, and 16 h (top panels). Heatmap of ChIP-seq reads density within 1kb around the center of distal enhancers, proximal enhancers, promoters, and random genomic regions at 0 h, 8 h, and 16 h (mid panels). Quantification of H3K27ac signals represented as bar plots (bottom panels). (B) Average H3K27ac signal profiles of active distal enhancers, proximal enhancers, and promoters (top panels). Quantification of H3K27ac signals represented as bar plots (bottom panels). (C) Average H3K27ac signal profiles for “consistently active” and “consistently inactive” states of distal enhancers, proximal enhancers, and promoters. Quantification of H3K27ac signals represented as bar plots. Numbers above plots indicate counts of elements for this condition. Data are presented as mean ± SEM. Statistical significance was assessed using paired non-parametric Friedman tests. ∗*p* < 0.05, ∗∗*p* < 0.01, ∗∗∗*p* < 0.001, ∗∗∗∗*p* < 0.0001.

Enhancers and promoters, as *CREs*, often regulate tumorigenesis and progression through changes in their activity.^23^ H3K27ac has been widely acknowledged as a hallmark of active enhancers and promoters.^24^ To investigate the activity of enhancers and promoters, we treated HepG2 cells with DOX at 0 h (pre-damage), 8 h (moderate damage), and 16 h (severe damage). Then we performed H3K27ac ChIP-seq to detect the activity of distal/proximal enhancers and promoters. Compared to random regions, the H3K27ac signal levels at both enhancers and promoters exhibited a bimodal distribution near their center, with a significant decrease followed by an increase (Figure 3A). Furthermore, promoter regions consistently exhibited significantly higher H3K27ac signal levels compared to distal/proximal enhancer regions (Figure S5). These findings suggest that after DNA damage suppression, enhancers and promoters both participate in the DDR process by increasing their activity.

To investigate whether the activity of enhancers and promoters is a critical factor for DDR, we classified all elements into active and inactive distal/proximal enhancers and promoters based on whether H3K27ac modification is present at the pre-damage period (0 h) (Table S4). We then analyzed the changes in H3K27ac levels in these regions following DNA damage. The results showed that H3K27ac levels of all types of active elements showed the same trend of change under DNA damage, that is, significantly decreased and then increased (Figure 3B). On the contrary, there was no significant change in H3K27ac levels for inactive elements during damage except for promoters (Figure S8).

To further compare the dynamic changes in H3K27ac levels of enhancer and promoter regions during DNA damage, we subdivided the pre-damage (0 h) active/inactive enhancers and promoters into two distinct categories: “consistently active” element regions, which are active at all time points (0 h, 8 h, and 16 h) and “consistently inactive” element regions, which are inactive at all time points (0 h, 8 h, and 16 h). The results showed that during the DNA damage process, most enhancers (distal: 68%; proximal: 75%) and promoters (87%) remained in a “consistently active” state during DNA damage (Table S4). Additionally, the H3K27ac signals of “consistently active” regions exhibited a significant decrease followed by a significant increase during DNA damage. On the contrary, there were no significant changes in the “consistently inactive” regions (Figure 3C). The previous results suggest a process where elements’ activity decreases after the initial stage of DNA damage, followed by a prompt increase of elements’ activity to resist the DNA damage. And this process primarily occurs in “consistently active” elements during DNA damage. In summary, after DNA damage suppression, enhancers and promoters both participate in the DDR process by increasing their activity. Furthermore, we found that the DDR predominantly occurs in regulatory elements that remain active.

### Enhancer and promoter regulatory network during DNA damage

**Figure 4:**
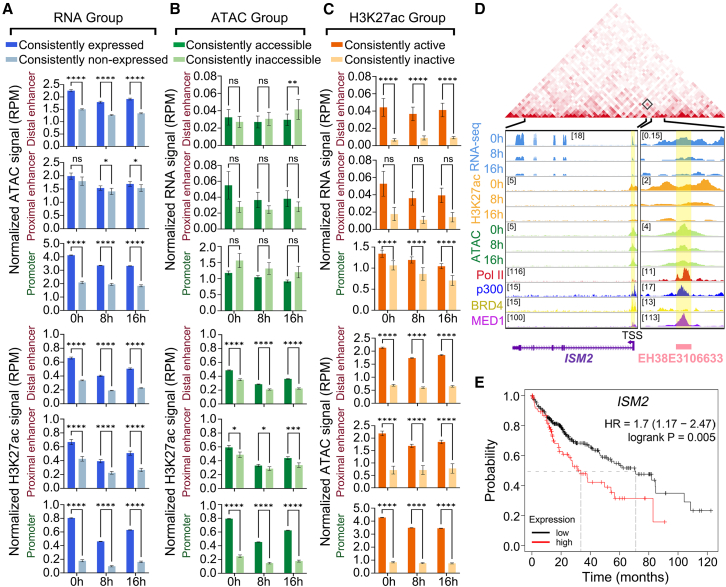
Dynamic relationships among expression, chromatin accessibility, and activity at distal/proximal enhancers and promoters during DNA damage, and regulation between the enhancer EH38E3106633 and target gene *ISM2* (A) Bar plots showing the chromatin accessibility and activity levels for distal/proximal enhancers and promoters in “consistently expressed” and “consistently non-expressed” states. (B) Bar plots showing the expression and activity levels for distal/proximal enhancers and promoters in “consistently accessible” and “consistently inaccessible” states. (C) Bar plots showing the expression and chromatin accessibility levels for distal/proximal enhancers and promoters in “consistently active” and “consistently inactive” states. Data are presented as mean ± SEM and analyzed using unpaired non-parametric Kolmogorov-Smirnov (K-S) test. ∗*p* < 0.05, ∗∗*p* < 0.01, ∗∗∗*p* < 0.001, ∗∗∗∗*p* < 0.0001. (D) Representation of Hi-C interactions (top panels) and genomic tracks (bottom panels) in the region containing the enhancer EH38E3106633 (chr14: 77502120–77502468) and *ISM2* gene (chr14: 77474394–77498816). The gray square labels the interaction between enhancer EH38E3106633 and *ISM2* promoter in the Hi-C interaction map. The enhancer EH38E3106633 and *ISM2* promoter region have been highlighted. (E) Kaplan-Meier survival curves are calculated for *ISM2* gene in HCC patients (*n* = 364). *p* value is calculated using log rank test.

Next, we investigated the dynamic relationships among expression, chromatin accessibility, and activity at distal/proximal enhancers and promoters during DNA damage. Firstly, we examined the chromatin accessibility and H3K27ac levels of enhancers and promoters in “consistently expressed” and “consistently non-expressed” states during the DNA damage. The results showed that, regardless of DNA damage or not, both ATAC and H3K27ac signals were significantly higher in “consistently expressed” distal enhancers and promoters compared to “consistently non-expressed” regions, suggesting these changes in distal enhancers and promoters do not have DNA damage specificity. Notably, for proximal enhancers, it is only under the post-damage period (8 h and 16 h) that their expression significantly affects accessibility (Figure 4A). This suggests that the expression of *CREs* play a critical role in influencing their chromatin accessibility and activity, and distinct DNA damage specificity is observed in proximal enhancers. Similarly, we analyzed the changes in expression and activity of enhancers and promoters in “consistently accessible” and “consistently inaccessible” states. The results indicate that at three time points (0 h, 8 h and 16 h), the H3K27ac signals were significantly higher in “consistently accessible” distal/proximal enhancers and promoters compared to “consistently inaccessible” regions, while there is no significant difference in expression levels (Figure 4B). This implies that enhancer and promoter chromatin accessibility is more closely related to their activity than to their expression levels during DNA damage, and expression of CREs often influences chromatin accessibility, whereas chromatin accessibility does not predominantly affect expression levels. Finally, we explored the changes in expression and chromatin accessibility levels of enhancers and promoters in “consistently active” and “consistently inactive” states. The results showed that both expression and chromatin accessibility levels were significantly higher in “consistently active” distal enhancers and promoters (except for proximal enhancers) compared to “consistently inactive” regions (Figure 4C). This indicates that high activity level of elements is often associated with high expression and high chromatin accessibility levels during DNA damage.

Enhancers typically perform their functions by positively regulating the expression of their target genes. Therefore, we identified 184 enhancer-gene pairs exhibiting dynamic coordinated changes in expression at three time points during DNA damage (Table S5). These enhancer-target gene pairs, with consistent expression changes, often play a critical role in HCC patients’ responses to DNA damage.^25^ For example, we found that the enhancer EH38E3106633 and the *ISM2* gene promoter exhibit high chromatin interaction, and they both showed a consistent downregulation of expression levels during DNA damage, which corresponds to our expectations of consistently downregulated ATAC and H3K27ac signals (Figure 4D). Previous research has shown that high expression of *ISM2* promotes tumor cell proliferation and migration in HCC patients.^26^ And survival analysis indicates that high expression of *ISM2* is associated with significantly lower survival times (Figure 4E), suggesting that DNA damage causes the downregulation of the *ISM2* gene in HCC by suppressing the expression of the enhancer EH38E3106633. Another example is the enhancer EH38E2303172 consistently upregulated the *ALB* gene following DNA damage. *ALB* is known to be crucial for maintaining HCC homeostasis,^27^ and survival analysis indicates that high expression of *ALB* is associated with significantly longer survival times in HCC patients (Figure S7).

### DNA damage leads to differential enrichment of the p53 transcription factor at enhancer regions

Previous researchers have found that TFs that binding to DNA damage sites can directly regulate the DDR process by altering chromatin structure,^10^ and the loss of activity of TFs can lead to DNA damage repair defects and causing tumorigenesis.^28^ To investigate the binding of TFs on enhancers and promoters in HepG2 cells during DNA damage, we performed TF footprinting analysis using the HINT-ATAC method. The results showed that the number of TF binding sites (TFBS) on enhancer and promoter regions initially decreased and then increased following DNA damage (Figure S8), which is consistent with changes of chromatin accessibility and H3K27ac levels observed in our results previously (Figures 2A and 3A).

**Figure 5:**
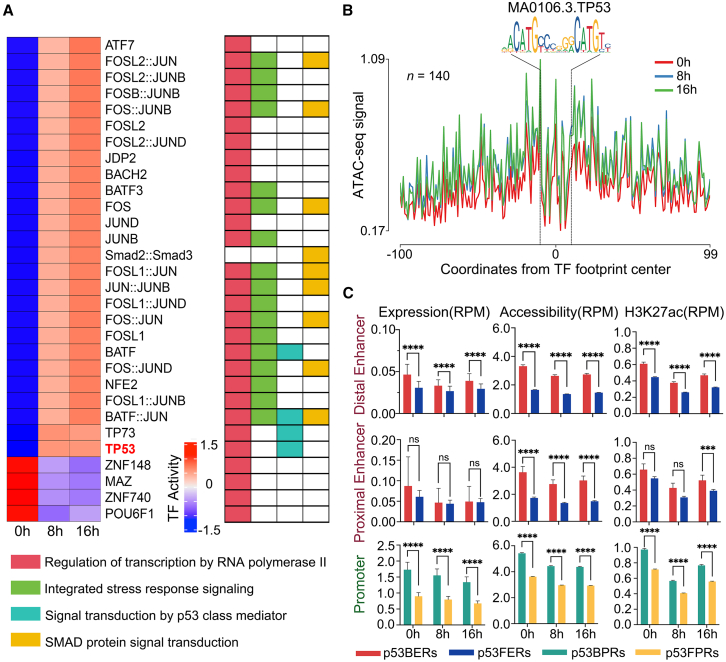
Transcription factor enrichment and p53 regulation during DNA damage (A) Heatmap showing TF footprint analysis of enhancer regions using HINT-ATAC software, highlighting the top30 TF footprints with significant differences in activity during DNA damage (*Z* score normalized) (left panels), and the double colon “::” represents a TF complex which contains two different TFs. Enrichment of the top30 TF footprints in GO BP pathways (right panels). (B) The footprint profile showing changes in the binding activity intensity of p53 in enhancer regions during DNA damage. Higher ATAC-seq signal indicates the higher binding activity of the TF. Numbers above plots indicate counts of TF footprints. (C) RNA-seq, ATAC-seq, and H3K27ac signals in p53BERs, p53FERs, p53BPRs, and p53FPRs during DNA damage. Data are presented as mean ± SEM and analyzed using unpaired non-parametric Kolmogorov-Smirnov (K-S) test. ∗*p* < 0.05, ∗∗*p* < 0.01, ∗∗∗*p* < 0.001, ∗∗∗∗*p* < 0.0001.

As TFs, they often perform their function through changes in their activity. Next, we quantified the TF binding activity in enhancer and promoter regions using ATAC-seq in HepG2 cells treated with DOX for 0 h, 8 h, and 16 h. The results showed that 501 TFs bound to enhancers and 551 TFs bound to promoters exhibited changes in TF binding activity after DNA damage (Table S6). To further identify the TFs with the most significant changes in binding activity, we, respectively, selected the top 30 TFs from enhancer and promoter regions for next analysis. Among them, 7 out of the top 30 TFs were commonly bound to both enhancer and promoter regions, including members of the JUN family (JUNB, JUND) and the FOS family (FOSL1, FOSL2), which are known to regulate cell proliferation, differentiation, and apoptosis in the NF-κB and Wnt pathways.^29^ We then performed GO BP enrichment analysis on the top 30 TFs bound to enhancers and promoters (Table S6). The results showed that the top 30 TFs in both enhancer and promoter regions were involved in “regulation of transcription by RNA polymerase II” and “integrated stress response signaling” pathways (Figures 5A and S9), indicating that DNA damage primarily affects TFs involved in transcriptional regulatory pathways. Particularly, the top 30 TFs in the enhancer regions uniquely participated in the “signal transduction by p53 class mediator” pathway in response to DNA damage and the “SMAD protein complex assembly” pathway related to cancer progression.^30^ Additionally, we found that p53 was present only in the top 30 TFs in enhancer regions, but was absent from the top 30 TFs in promoter regions (Figures 5A and S9). This result is similar to previous reports that p53 tends to bind to regions upstream of the TSS rather than the promoter region.^31^ To further investigate whether there is an observably difference in p53 binding activity between enhancer and promoter regions, we quantified p53 binding activity using ATAC-seq and visualized the changes in p53 binding activity during DNA damage. We found that while the p53 binding activity at enhancers was significantly upregulated during DNA damage, its binding activity at promoters remained unchanged (Figures 5B and S10). Our results suggest that p53 primarily exerts its function through binding activity changes in enhancer regions rather than promoter regions under DNA damage.

To further elucidate the regulatory effects of p53 on enhancers and promoters during DNA damage, based on whether p53 binds to the element or not, we divided enhancer regions into p53-bound (p53BERs) and p53-free enhancer regions (p53FERs), and similarly, promoter regions into p53-bound (p53BPRs) and p53-free promoter regions (p53FPRs) (Table S7). First, we investigated the impact of p53 on the expression levels of enhancers and promoters during DNA damage. The results showed that, except for proximal enhancers, regardless of DNA damage, the expression levels in distal p53BERs and p53BPRs were significantly higher than those in distal p53FERs and p53FPRs, suggesting that the effect of p53 on the expression levels of regulatory elements is independent of DNA damage and exhibits regulatory element specificity (Figure 5C). Similarly, we assessed the impact of p53 on the chromatin accessibility of enhancer and promoter regions during DNA damage. The results showed that the chromatin accessibility levels in both distal/proximal p53BERs and p53BPRs were significantly higher than those in distal/proximal p53FERs and p53FPRs during DNA damage (Figure 5C). Finally, we investigated how p53 influences H3K27ac levels at enhancers and promoters in response to DNA damage. As expected, consistent with previous reports,^32^ our results also showed that, regardless of DNA damage, H3K27ac levels in distal p53BERs, proximal p53BERs (only in 16 h) and p53BPRs were significantly higher than those in p53FERs and p53FPRs (Figure 5C). In summary, we found TFs with significant activity changes during DNA damage, which are bound to enhancers and promoters, predominantly influencing transcriptional regulatory pathways. And DNA damage leads to the differential enrichment of p53 in enhancer regions rather than promoter regions. We also found that during the DNA damage process, the binding of p53 results in a significant increase in the expression levels, chromatin accessibility, and activity in distal enhancer and promoter regions.

### Conclusion

This study integrated RNA-seq, ATAC-seq, and H3K27ac ChIP-seq data to systematically analyze the dynamic changes of *CREs* (enhancers and promoters) in HepG2 cells during the DNA damage process. Our findings demonstrate that distal enhancers exhibited an initial decrease in expression, accessibility, and activity at 8 h, followed by a significant rebound at 16 h as DNA damage intensified. In contrast, the expression of promoter regions continuously decreased, indicating distinct regulatory characteristics. Furthermore, the pre-damage accessibility and activity states of enhancer and promoter regions significantly influenced their dynamic behavior post-damage. Additionally, we identified p53-mediated differential regulation of enhancers versus promoters: following DNA damage, p53 binding activity was markedly upregulated in enhancer regions, while it remained unchanged in promoter regions. Based on these findings, we propose a temporal chromatin remodeling model for regulatory elements in HepG2 cells during DNA damage: in the early phase of DNA damage, chromatin compaction in both enhancer and promoter regions induces global transcriptional suppression; in the later phase, distal enhancer regions undergo chromatin reopening, accompanied by increased expression of eRNAs, which subsequently activate their target genes involved in DNA damage repair. Overall, our study reveals the complex dynamic changes within enhancers and promoters in HepG2 cells during DNA damage, providing novel insights into the molecular mechanisms underlying the DDR in HCC.

## Resource availability

### Lead contact

Further information and requests for resources should be directed to and will be fulfilled by the lead contact, Zhiyun Guo (zhiyunguo@swjtu.edu.cn).

### Materials availability

This study did not generate new unique materials.

### Data and code availability

• All raw sequencing and processed data generated in this study, including RNA-seq (GSE278054), ATAC-seq (GSE278055), and ChIP-seq (GSE278056), are deposited in the Gene Expression Omnibus (GEO) database.
• Any additional information required to reanalyze the data reported in this paper is available from the lead contact upon request.

## Acknowledgments

We thank the Informatization of Southwest Jiaotong University for providing technical support. This work was supported by the Key Research and Development Project of Sichuan Science and Technology Program (24ZDYF0889), Basic Research Cultivation Support Program of 10.13039/501100012226Fundamental Research Funds for the Central Universities (2682023ZTPY071) and Sichuan Science and Technology Program under grant (2022NSFSC0779).

## Author contributions

Conceptualization, Z.G.; methodology, J.Z., T.M., and L.X.; formal analysis, J.Z., T.M., and L.X.; investigation, J.Z., T.M., and L.X.; resources, J.Z., T.M., and L.X.; writing – original draft, J.Z., T.M., and Z.G.; writing – review and editing, J.Z., T.M., and Z.G.; supervision, Z.G.; funding acquisition, Z.G.

## Declaration of interests

The authors declare no competing interests.

## STAR★Methods

### Key resources table

**Table:** 

REAGENT or RESOURCE	SOURCE	IDENTIFIER
**Antibodies**		

p53 primary antibody	SAB	Cat# 38007
β-actin primary antibody	SAB	Cat# 21800
HRP-goat anti-mouse secondary antibody	Beyotime	Cat# A0216

**Chemicals, peptides, and recombinant proteins**		

Doxorubicin	Sigma-Aldrich	Cat# D1515

**Critical commercial assays**		

Fragment Analyzer 5400	Agilent Technologies	NA
NEBNext® UltraTM RNA Library Prep Kit	NEB (New England Biolabs)	Cat# E7530L
Illumina NovaSeq 6000	Illumina	NA
NanoPhotometer® spectrophotometer	IMPLEN	NA
Qubit® 3.0 Fluorometer	Life Technologies	Cat# Q33216
Qubit® DNA Assay Kit	Life Technologies	Cat# Q32851
Agilent Bioanalyzer 2100	Agilent Technologies	Cat# G2939BA

**Deposited data**		

RNA-seq	This paper	GEO: GSE278054
H3K27ac ChIP-seq	This paper	GEO: GSE278056
ATAC-seq	This paper	GEO: GSE278055
Enhancer and Promoter regions	ENCODE	ENCSR356UJZ
*in situ* Hi-C	ENCODE	ENCFF050EKS
Pol II ChIP-seq	ENCODE	ENCFF425QWO
EP300 ChIP-seq	ENCODE	ENCFF962CGI
BRD4 ChIP-seq	ENCODE	ENCFF863BZH
MED1 ChIP-seq	ENCODE	ENCFF870CZP
TP53 ChIP-seq	ENCODE	ENCFF701DTE

**Experimental models: Cell lines**		

HepG2	the National Collection of Authenticated Cell Cultures	Cat# CSTR:19375.09.3101HUMSCSP510

**Software and algorithms**		

HISAT2	Kim et al.^33^	http://daehwankimlab.github.io/hisat2/
DeepTools	Ramírez et al.^34^	https://github.com/deeptools/deepTools
FeatureCounts	Liao et al.^35^	https://subread.sourceforge.net/featureCounts.html
StringTie	Pertea et al.^36^	https://github.com/gpertea/stringtie
DESeq2	Love et al.^37^	https://bioconductor.org/packages/release/bioc/html/DESeq2.html
biomaRt	Durinck et al.^38^	https://bioconductor.org/packages/release/bioc/html/biomaRt.html
BEDTools	Quinlan et al.^39^	https://bedtools.readthedocs.io/en/latest/index.html
ChIPSeeker	Yu et al.^40^	https://bioconductor.org/packages/release/bioc/html/ChIPseeker.html
Bowtie2	Langmead et al.^41^	https://bowtie-bio.sourceforge.net/bowtie2/index.shtml
MACS2	Qian et al.^42^	https://github.com/macs3-project/MACS/wiki
GREAT	McLean et al.^43^	http://great.stanford.edu/public/html/
WashU Epigenome Browser	Li et al.^44^	http://epigenomegateway.wustl.edu
HINT-ATAC	Li et al.^45^	https://reg-gen.readthedocs.io/en/latest/hint/introduction.html

### Experimental model and study participant details

#### Tumour cell line

HepG2 cells were cultured in DMEM (Gibco, USA) supplemented with 10% fetal bovine serum (FBS) and 1% penicillin–streptomycin.

### Method details

#### Cell culture and DOX treatment

When the HepG2 cells spreading rate reached 80%, the cells were cultured with complete medium containing DOX at a final concentration of 0.5μg/mL. Subsequently, the cells were treated for various durations (0, 4, 8, 12, 16, 24, 36, and 48h) before samples were extracted for additional experimental validation.

#### Western blotting

In short, treat HepG2 cells with DOX and collect cells at 0, 4, 8, 12, 16, 24, 36, and 48h. Subsequently, lyse the cells with RIPA lysis buffer and quantify total protein using the BCA kit. Then, separate proteins by SDS-PAGE gel and transfer onto PVDF membrane (Millipore, Bedford, MA, USA). After blocking the PVDF membrane with 5% skim milk powder, incubate with primary antibodies p53 (SAB, #38007) or β-actin (SAB, #21800). followed by incubation with secondary antibody HRP-goat anti-mouse (Beyotime, A0216). Finally, visualize the protein bands using the BeyoECL Plus kit and the ChemiDocXRS system (BioRad, USA).

#### RNA-seq

HepG2 cells treated with DOX for 0h, 8h, and 16h were used as sequencing samples. The RNA integrity was assessed using the Fragment Analyzer 5400 (Agilent Technologies, CA, USA). Total RNA was used as the input material for RNA sample preparation. Sequencing libraries were generated using the NEBNext® UltraTM RNA Library Prep Kit for Illumina® (NEB, USA) according to the manufacturer’s instructions, and sequencing was performed on the Illumina NovaSeq 6000 platform, producing 150 bp paired-end reads.

#### Identification of distal/proximal enhancers and promoters

The candidate enhancer and promoter regions BED file for HepG2 cells ("ENCSR356UJZ") was downloaded from the ENCODE.^46^ Using bedtools (v2.30.0),^39^ enhancers overlapping known coding regions (extended 1 kb from the transcription start site and transcription end site) and all blacklist regions were removed, following previous studies.^25^ Proximal enhancers were defined as those located between 1 kb and 2 kb upstream of the gene TSS, and distal enhancers were defined as those located more than 2 kb upstream of the gene TSS. The information regarding the +/- strands of the promoter sequences was annotated using the R package "ChIPseeker".^40^

#### RNA-seq analysis of enhancers and promoters

Raw fluorescence image files were obtained from the Illumina platform and converted into short reads (raw data) through base calling, recorded in FASTQ format, which includes sequence information and corresponding sequencing quality information. The HISAT2 (v2.1.0)^33^ software was used to build the index of the reference genome (hg38) and perform sequence alignment of the RNA-seq data with default parameters. The multiBigwigSummary (v3.5.1)^34^ software was used to quantify enhancer RNA and promoter RNA, and the RPM of RNA-seq within the enhancer and promoter regions were selected as a measure of their expression levels. eRNAs and pancRNAs with RPM>0 at without treatment group (0h) were defined as the expressed enhancers and promoters. Unless otherwise specified, all profile plots and heatmaps in the text are generated using deepTools.^34^

#### Gene expression and functional enrichment analysis

FeatureCounts (v2.0.3)^35^ software was used to quantify the RNA-seq read counts of genes, with parameters (-p --countReadPairs -O). StringTie (v1.3.3)^36^ software was utilized to normalize the read counts of genes, and the resulting transcripts per million (TPM) were used as indicators of gene expression levels. Genes with TPM≥1 at 0h, 8h, and 16h were defined as the expressed group. Differential analysis was performed using the R package "DESeq2",^37^ with filtering criteria (|log2FoldChange|>1, FDR<0.05). The R package "biomaRt"^38^ was used to annotate the filtered differentially expressed genes (DEGenes). Enrichment analysis was conducted using the R package "clusterProfiler", and graphical representations, including volcano plots, were created using the R package "ggplot2". RNA-seq data visualization was achieved using bamCoverage (v3.5.1)^34^ software with parameters (--normalizeUsing CPM --binSize 50 --smoothLength 60).

#### ATAC-seq

HepG2 cells treated with DOX for 0h, 8h, and 16h were used as ATAC-seq sequencing samples. Nuclei were extracted from the samples and resuspended in the Tn5 transposase reaction mixture. The transposition reaction was incubated at 37°C for 30 minutes. After transposition, equal amounts of Adapter1 and Adapter2 were added, followed by PCR amplification of the library. Post-PCR, the library was purified using AMPure beads and its quality was assessed using Qubit. Indexed samples were clustered on the cBot Cluster Generation System using the TruSeq PE Cluster Kit v3-cBot-HS (Illumina). After cluster generation, sequencing was performed on the Illumina platform, producing 150 bp paired-end reads.

#### Chromatin accessibility analysis of enhancers and promoters

Trimming was performed using fastp (v0.20.0) to obtain clean reads. Bowtie2 (v2.5.0)^41^ was then used to construct an index of the reference genome (hg38), and sequences were aligned with parameter (--no-unal). Samtools (v1.16.1) was employed to retain reads with MAPQ≥30, with parameters (-f 2 and -q 30), and PCR duplicates were removed using Picard. Following the methods outlined in the ENCODE-ATAC-Seq pipeline (https://github.com/ENCODE-DCC/atac-seq-pipeline), BAM files were converted to tagAlign format. The multiBigwigSummary (v3.5.1) software was used to quantify the chromatin accessibility levels of enhancer and promoter regions. Peak calling was conducted using MACS2 (v2.2.7.1)^42^ with parameters (-f BED -q 0.05 -B -g hs --nomodel --shift -100 --extsize 200 --keep-dup all --SPMR). ATAC-seq peak regions at three time points (0h, 8h, and 16h) were overlapped with enhancer and promoter regions, defining enhancers and promoters overlapping with 0h peaks as accessible enhancer/promoter and non-overlapping ones as inaccessible enhancer/promoter. As a control group for these regulatory element regions, using bedtools (v2.30.0), 10,000 regions with 2kb were randomly selected across the whole genome and designated as "random regions". Additionally, "random accessible regions" were defined as follows: regions were randomly selected from the ATAC peaks, the length of random accessible regions are determined by peaks, excluding those overlapping with enhancer and promoter regions.

#### H3K27ac ChIP-seq

HepG2 cells treated with DOX for 0h, 8h, and 16h were used as sequencing samples. First, the samples were crosslinked by adding the final concentration of 1% formaldehyde solution to the culture medium and incubating at room temperature for 10 minutes. Crosslinking was then terminated with the final concentration of 0.125M glycine solution. After crosslinking, cells underwent H3K27ac ChIP-seq. Briefly, DNA degradation and contamination in the ChIP samples were monitored on agarose gels. DNA purity was assessed with the NanoPhotometer® spectrophotometer (IMPLEN, CA, USA). DNA concentration was measured with the Qubit® 3.0 Fluorometer (Life Technologies, CA, USA) using the Qubit® DNA Assay Kit. Purified DNA was used for ChIP-seq library preparation. The samples were then subjected to paired-end sequencing on the Illumina platform (Illumina, CA, USA). Library quality was evaluated on the Agilent Bioanalyzer 2100 system.

#### H3K27ac analysis of enhancers and promoters

Like ATAC-seq analysis method, sequence alignment was performed using Bowtie2 (v2.5.0). Samtools (v1.16.1) and Picard were used to remove low-quality reads and PCR duplicates. Visualization of the ChIP-seq data was achieved using bamCompare (v3.5.1) with parameters (--normalizeUsing CPM --operation subtract --binSize 50 --smoothLength 60). The multiBigwigSummary (v3.5.1) software was used to quantify the H3K27ac levels of enhancer and promoter regions. Peak calling was conducted using MACS2 (v2.2.7.1) with parameters (-f BED -q 0.05 -B -g hs --keep-dup all --SPMR). ChIP-seq peak regions at three time points (0h, 8h, and 16h) were overlapped with enhancer and promoter regions, defining enhancers and promoters overlapping with 0h peaks as active enhancer/promoter and non-overlapping ones as inactive enhancer/promoter.

#### Identification of enhancer and promoter regions with consistent trend changes

Enhancer and promoter regions with consistent trend changes across three omics features were defined as follows: Enhancers and promoters with RNA expression levels RPM>0 at all three time points (0h, 8h, and 16h) were defined as the "consistently expressed" group; those with RPM=0 at all three time points (0h, 8h, and 16h) were defined as the "consistently non-expressed" group. Enhancers and promoters overlapping with ATAC/H3K27ac peaks at all three time points (0h, 8h, and 16h) were defined as the "consistently accessible/active" group; those not overlapping at any time point (0h, 8h, and 16h) were defined as the "consistently inaccessible/inactive" group.

#### Enhancer target gene identification and survival analysis

Identification of enhancer target genes involved two approaches following the previous methods^47^: First, the "GREAT"^43^ tool was used to apply the nearest gene model for matching enhancers to genes within their regulatory regions. Second, *in situ* Hi-C experimental data for HepG2 cells from the ENCODE (ENCFF050EKS) were utilized to identify long-range interactions between enhancers and target genes. And Pol Ⅱ (ENCFF425QWO), p300 (ENCFF962CGI), BRD4 (ENCFF863BZH) and MED1 (ENCFF870CZP) were downloaded. Multi-omics signal tracks were visualized using the "WashU Epigenome Browser".^44^ Survival analysis was performed using the R packages "survival" and "survminer", with clinical data for HCC patient samples obtained from the TCGA database.

#### Transcription factors footprint analysis

To identify TFs with differential footprints in enhancer and promoter genomic regions, ATAC-seq BAM files from 0h, 8h, and 16h were used as input for HINT-ATAC (v1.0.2)^45^ to compute TF footprint information across the genome. Subsequently, bedtools was used to extract TF footprints within enhancer and promoter regions. The protection score (which corresponds to the difference in cleavage events between the footprint and flanking regions), along with the Tag Count (TC) value in HINT-ATAC, were used to assess differential TF binding activity. We then calculated the standard deviation of TF binding activity at three time points (0h, 8h, and 16h) and ranked the TFs in descending order of standard deviation, selecting the top 30 as the highly variable TFs for further analysis.

#### Identification of p53 protein binding enhancers and promoters

The p53 ChIP-seq data for the HepG2 cell line were downloaded from the ENCODE (ENCFF701DTE). Enhancer and promoter regions overlapping with p53 peaks were defined as p53-bound enhancer regions (p53BERs) and p53-bound promoter regions (p53BPRs), respectively. Enhancer and promoter regions not overlapping with p53 peaks were defined as p53-free enhancer regions (p53FERs) and p53-free promoter regions (p53FPRs).

#### Statistical analysis

All assumptions for statistical tests (e.g. normality and homogeneity for parametric tests) were formally tested. Paired non-parametric Friedman test or unpaired non-parametric Kolmogorov-Smirnov (K-S) test were used to assess statistical significance. The *p*-value of less than 0.05 was considered significant for all tests (∗ *P*<0.05; ∗∗ *P*<0.01; ∗∗∗ *P*<0.001; ∗∗∗∗ *P*<0.0001).

### Additional resources

This study did not generate additional data.

## Supplemental information

Document S1. Figures S1–S10

Table S1. The expression of enhancers and promoters at 0h, 8h, 16h based on RNA-seq, related to Figure 1

Table S2. The differentially expressed genes from 0h to 16h and genes responding to DNA damage pathways were identified, related to Figure 1

Table S3. The accessibility of enhancers and promoters at 0h, 8h, 16h based on ATAC-seq, related to Figure 2

Table S4. The activity of enhancers and promoters at 0h, 8h, 16h based on H3K27ac ChIP-seq, related to Figure 3

Table S5. Enhancer-gene pairs were identified using the "GREAT" tool and combined with data from situ Hi-C, related to Figure 4

Table S6. HINT-ATAC software was used to discover differential TF footprints in regions of enhancer and promoter in HepG2, related to Figure 5

Table S7. p53-bound enhancer regions and p53-free enhancer regions; p53-bound promoter regions and p53-free promoter regions were identified, related to Figure 5
