# Supplementary material for: Dynamic properties of enhancer and promoter during DNA damage in hepatocellular carcinoma
Source: iScience. 2025 May 2;28(6):112565. doi: 10.1016/j.isci.2025.112565 (PMC12178793; doi:10.1016/j.isci.2025.112565)
Supplement: Document S1. Figures S1–S10 [file mmc1.pdf]

iScience, Volume *28*

Supplemental information

Dynamic properties of enhancer and promoter during DNA damage in hepatocellular carcinoma

Jinyuan Zhang, Tianyu Ma, Longjun Xian, and Zhiyun Guo

Supplementary Figures

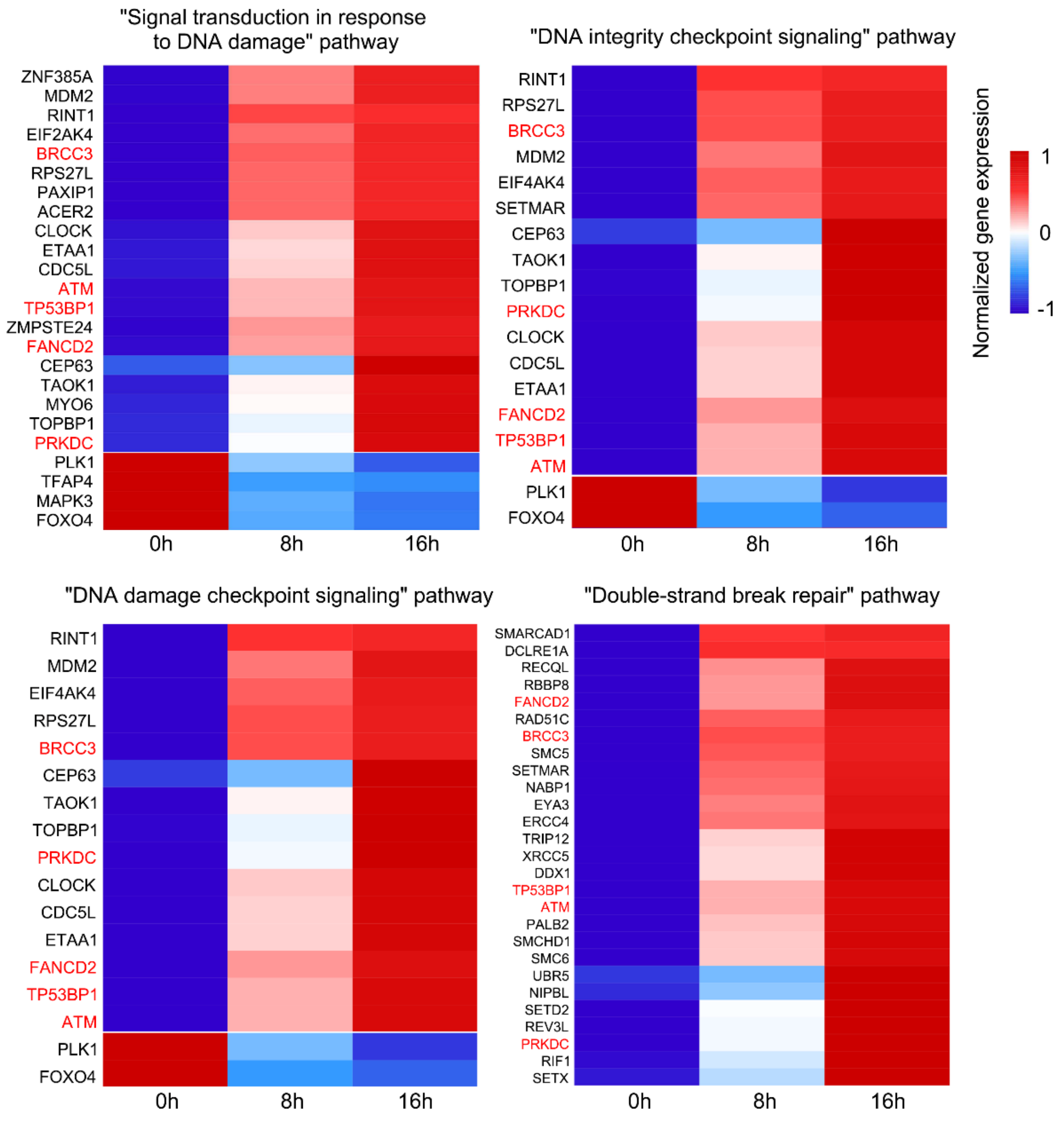

Figure S1 Heatmap showing the expression of genes involved in "Signal transduction in response to DNA damage", "DNA damage checkpoint signaling", "Double-strand break repair" and "DNA integrity checkpoint signaling" pathways. The identical genes that are present in the four DNA damage-related pathways have been highlighted in red.

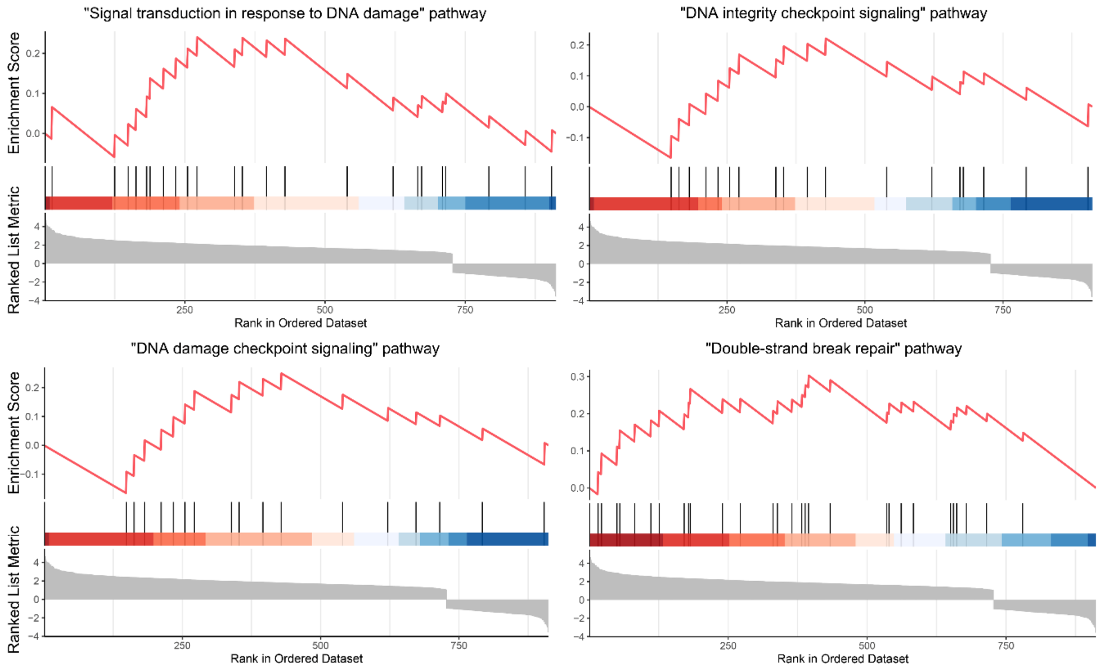

Figure S2 GSEA enrichment map of consistency of up-regulated or down-regulated genes in the "Signal transduction in response to DNA damage", "DNA damage checkpoint signaling", "Double-strand break repair" pathway and "DNA integrity checkpoint signaling".

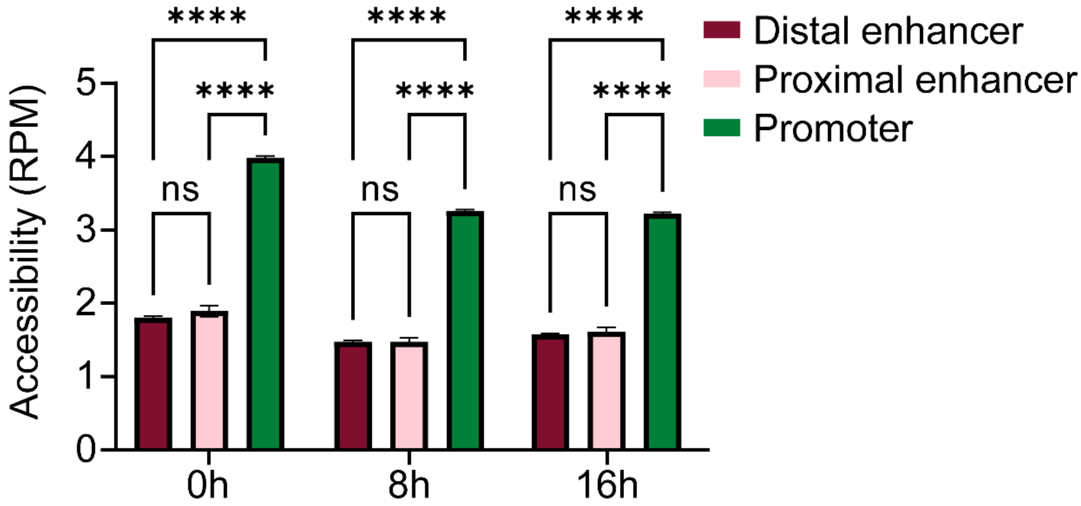

Figure S3 Bar plots of ATAC-seq signal comparison among distal enhancers, proximal enhancers and promoters. Data are presented as mean ± SEM and analyzed using unpaired non-parametric Kolmogorov-Smirnov (K-S) test. *p<0.05, **p<0.01, ***p<0.001, ****p<0.0001. Supplementary to Figure 2A.

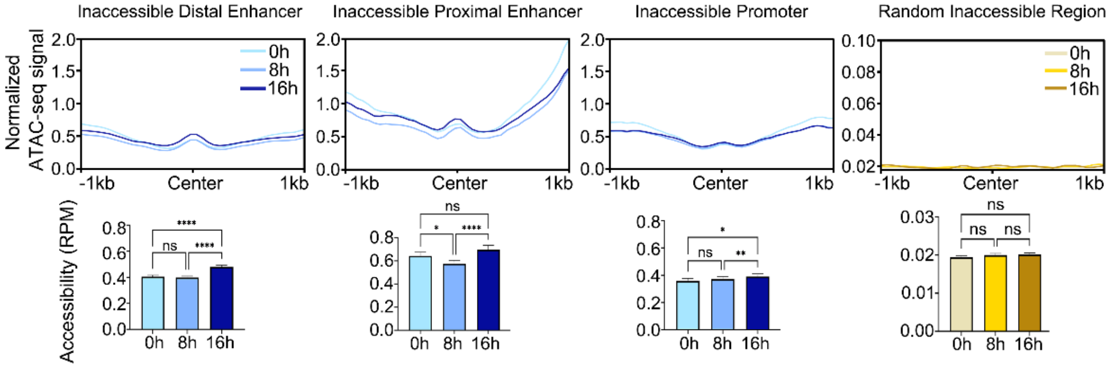

Figure S4 Average ATAC-seq signal profiles of inaccessible distal enhancers, proximal enhancers, promoters, and random inaccessible regions (top panels). Quantification of ATAC-seq signals represented as bar plots (bottom panels). Supplementary to Figure 2B.

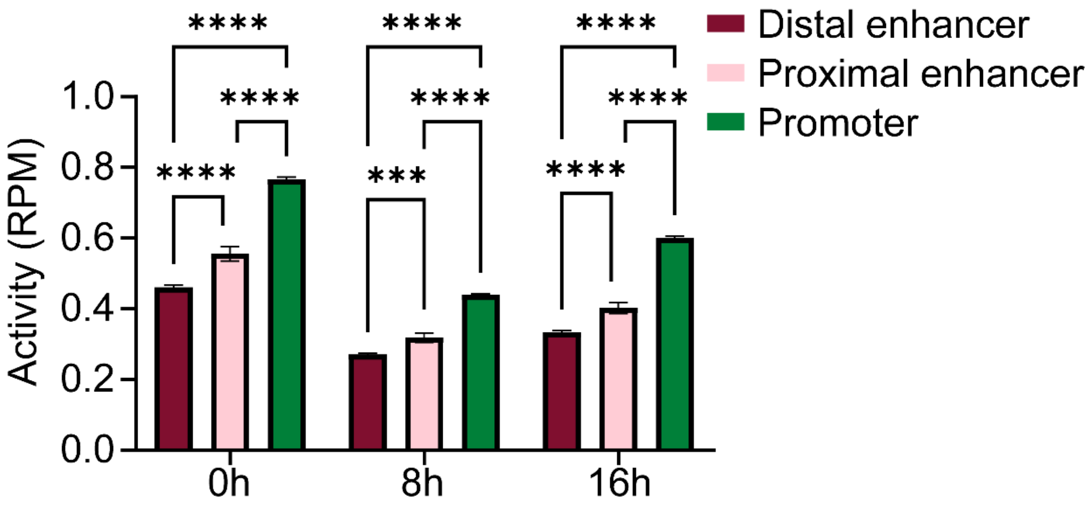

Figure S5 Bar plots of H3K27ac signal comparison among distal enhancers, proximal enhancers and promoters. Data are presented as mean ± SEM and analyzed using unpaired non-parametric Kolmogorov-Smirnov (K-S) test. *$p<0.05$, **$p<0.01$, ***$p<0.001$, ****$p<0.0001$. Supplementary to Figure 3A.

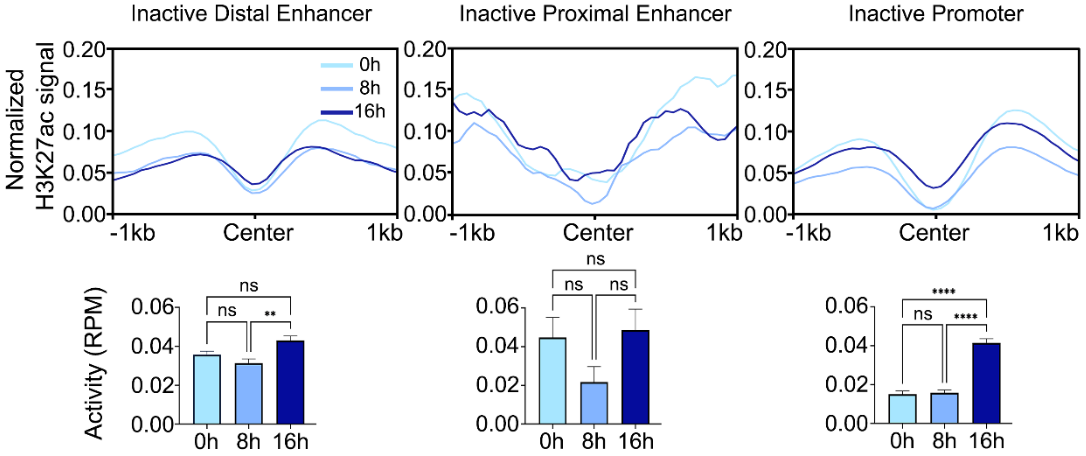

Figure S6 Average H3K27ac signal profiles of inactive distal enhancers, proximal enhancers and promoters (top panels). Quantification of H3K27ac levels represented as bar plots (bottom panels). Supplementary to Figure 3B.

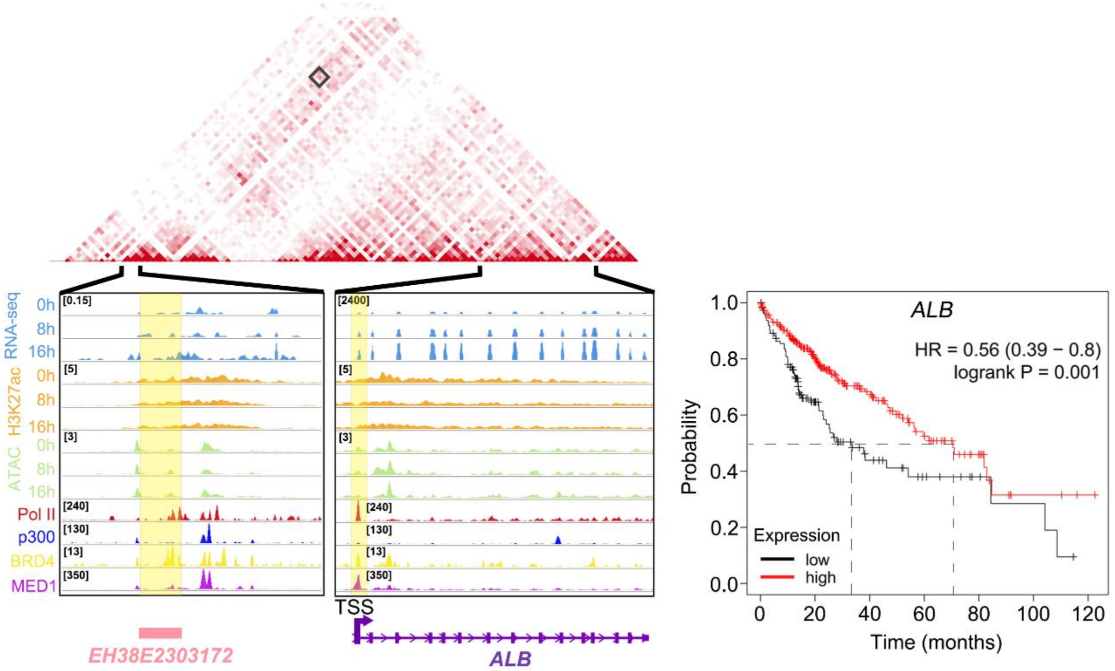

Figure S7 Representation of Hi-C interactions and genomic tracks in the region containing the enhancer EH38E2303172 (chr4: 73341452-73341706) and *ALB* gene (chr4: 73397114-73421482). The enhancer EH38E2303172 and *ALB* promoter region have been highlighted. Kaplan-Meier survival curves are calculated for *ALB* gene in HCC patients (n=364). P value is calculated using log-rank test.

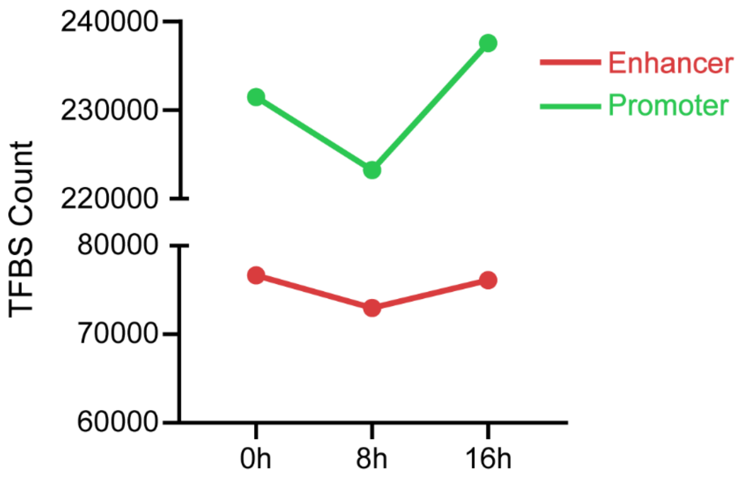

Figure S8 Line diagram of the number of TFBS in enhancer and promoter regions during DNA damage.

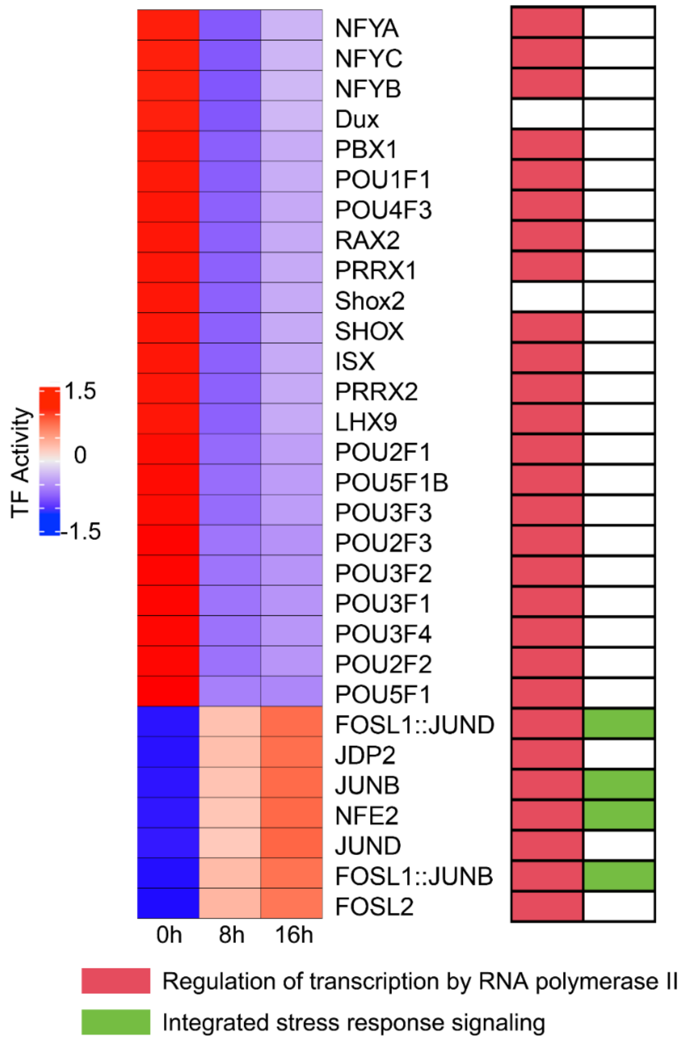

Figure S9 Enrichment plot depicts the Top30 TFs footprinting with significant changes (Z-score normalized) in promoter regions during DNA damage.

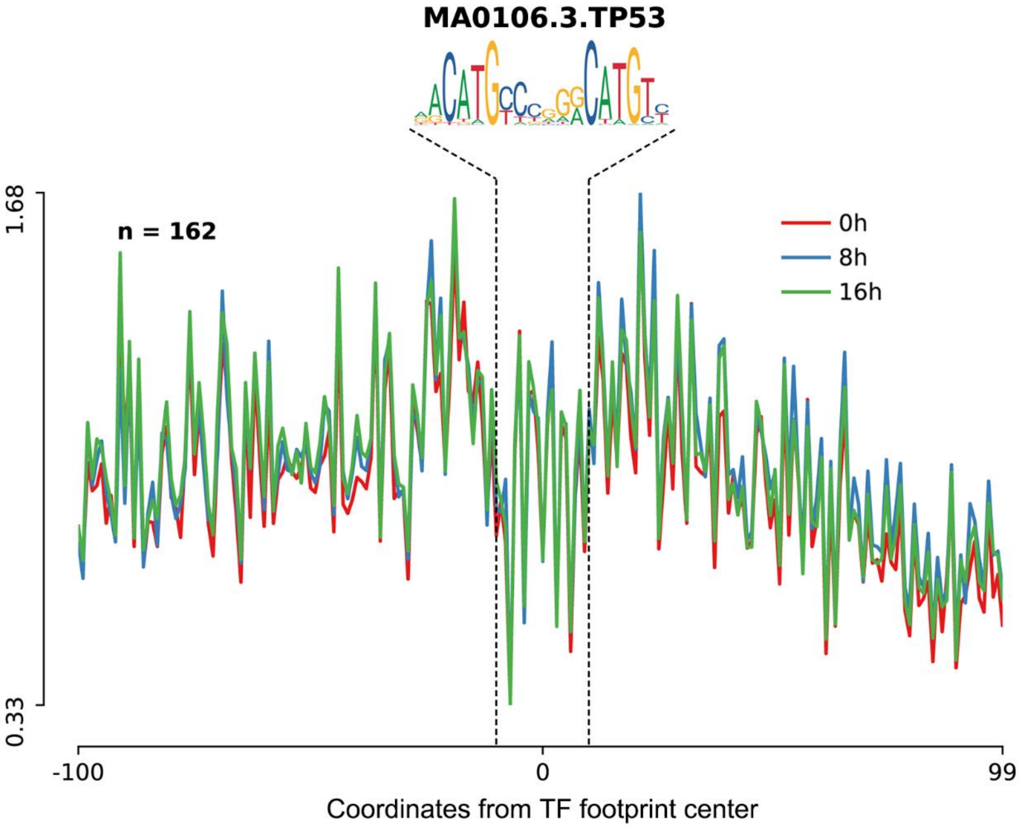

Figure S10 The footprint profile of Changes of p53 footprint in promoter regions after DNA damage. Numbers above plots indicate counts of TF footprints.
